# Frameworks Used to Engage Postsecondary Students in Campus Mental Health Research: A Scoping Review

**DOI:** 10.1111/hex.70144

**Published:** 2025-03-21

**Authors:** Kristin Cleverley, Soha Salman, Julia Davies, Lexi Ewing, Emma McCann, Katherine Sainsbury, Mikaela Gray, Carrie K. Y. Lau, Orly Lipsitz, Sapolnach Prompiengchai

**Affiliations:** ^1^ Lawrence S. Bloomberg Faculty of Nursing and Department of Psychiatry, Faculty of Medicine University of Toronto Toronto Canada; ^2^ Centre for Addiction and Mental Health Toronto Canada; ^3^ Lawrence S. Bloomberg Faculty of Nursing University of Toronto Toronto Canada; ^4^ Gerstein Science Information Centre University of Toronto Toronto Canada; ^5^ Factor‐Inwentash Faculty of Social Work University of Toronto Toronto Canada; ^6^ Department of Psychological Clinical Science University of Toronto Toronto Canada; ^7^ Department of Psychology University of Toronto Scarborough Toronto Canada

**Keywords:** mental health, postsecondary students, research engagement

## Abstract

**Background:**

There is an increasing prevalence of mental health concerns reported among postsecondary students (PSS) and growing demands for care on campuses around the world, as such there is an urgent need for research and innovations in PSS mental health that engages PSS. However, best practices and guidelines for facilitating PSS engagement in research is lacking. To address this gap, we undertook this review to explore frameworks used for engaging with PSS in research focused on PSS mental health.

**Methods:**

A scoping review of the academic literature was conducted. Frameworks used to engage PSS in mental health research were identified and categorized using the taxonomy of patient and public engagement by Greenhalgh et al. A list of barriers and facilitators to engaging with PSS was also identified and reported.

**Results:**

Of the articles assessed for full‐text screening (*n* = 167), 26 journal articles were included. Frameworks used for engaging PSS in mental health research were classified into one of the three categories from Greenhalgh et al.'s taxonomy: study‐focused (*n* = 14), partnership‐focused (*n* = 9) and power‐focused (*n *= 3). No relevant frameworks were found for two categories: priority‐ and report‐focused. Seven documents reported relational or process‐related barriers and/or facilitators to engaging with PSS. Based on these findings, recommendations were drafted with PSS advisors on how to implement an engagement framework in PSS mental health research.

**Conclusions:**

We identified existing practices outlined within frameworks used to engage PSS and barriers and facilitators to engage with PSS in mental health research. Based on the review findings and PSS advisors recommendations, a need for developing a comprehensive engagement framework specific to the PSS context was identified.

**Patient or Public Contribution:**

The research team led consultations with a PSS advisory group for this review. Student advisors were actively engaged in data analysis, which included categorizing and drafting of recommendations, and the preparation of this manuscript.

## 1 Introduction

There is growing concern about student mental health at postsecondary institutions (e.g., universities and colleges), with increasing rates of psychological distress, mental health diagnoses and mental health service seeking among students seen over the past decade [1, 2]. A recent systematic review and meta‐analysis found that the current prevalence of anxiety and depression symptoms among postsecondary students (PSS) are 39% and 33% respectively [3]. Poor mental health contributes to impairment across multiple domains of a student's life, including decreased academic performance, difficulty obtaining future employment, and challenges maintaining interpersonal relationships, in addition to further exacerbating existing concerns (e.g., onset of suicidal thoughts and behaviours) and worsening quality of life [4, 5, 6, 7, 8]. The COVID‐19 pandemic has only introduced further challenges to PSS mental health, with longitudinal studies identifying increased anxiety, depression and substance use among PSS over the course of the pandemic [9]. As a result, there is increasing demand for postsecondary campuses to develop and implement mental health interventions and care frameworks that include student‐centred policies and evidence‐based practices to support PSS mental health [10, 11]. Postsecondary campuses are increasingly being advised to prioritize continuous improvement of their student mental health approach through conducting evaluations of existing services, as well as leading research to advance understanding of how to support student mental health [10, 11]. A key component of the current call to action is to partner with PSS in knowledge production and in the design, implementation and evaluation of interventions [10], making meaningful engagement with PSS in campus mental health research an essential pillar to responding to the current mental health crisis [12]. This reflects current recognition in the wider health research community of the necessity and importance of partnering with, and engaging, persons with lived experience in research (typically termed ‘patient engagement’) [13, 14]. As experts in their own mental health experiences and needs, PSS have a similarly crucial role in research being conducted on their mental health and on campus‐based mental health programmes and services.

Patient engagement refers to the active collaboration with patients (or persons with lived experience of a particular health issue) in conducting research, where patients' voices are integrated throughout the research process, from developing research questions to disseminating findings [15]. It is increasingly viewed as the right of healthcare users with lived experience to colead health research, given that they have the highest stake in the impacts of research findings on policies and clinical practices [15, 16]. In particular, the engagement of patients, or persons with lived experience, in mental health research has positive impacts on patient partners, researchers and research participants [13]. Meaningful engagement provides unique expertise which can lead to more rigorous and feasible mental health research design with improved procedures (e.g. ethics protocols, participant recruitment and retention and data collection) as well as increasing the credibility, uptake and utilization of research findings [13]. As a result, patient engagement is being prioritized by researchers and national research funding agencies globally [15, 16, 17]. While beneficial to the design, operation and impact of research studies, meaningful patient engagement can be challenging due to barriers such as time constraints [18, 19, 20, 21], difficulties with planning [18, 22], limited guidelines [23, 24] and funding opportunities available for facilitating engagement [19, 21, 22]. Improperly planned or poorly supported patient engagement can lead to harms such as tokenism, exacerbation of power imbalances within the research team, or negative emotional impacts on patient partners [25], highlighting the importance of fully understanding the supporting principles of this practice, as well as potential barriers and facilitators, before embarking on patient‐engaged research. Frameworks or models present a useful tool to support the practical implementation of the principles of patient engagement. Several scoping and systematic reviews exist which have catalogued and assessed frameworks and models to support patient engagement [26, 27, 28], including for specific populations [21, 29], and these serve as an important resource for researchers looking to develop a plan to meaningfully engage patient partners in their research.

However, to our knowledge, there has been no evaluation of frameworks used to engage PSS in mental health research. PSS are a unique, yet diverse, population which may include youth moving away from home for the first time, mature students with caregiving responsibilities, veterans, or international students, among many other groups, learning in undergraduate, professional, or graduate level programmes [30, 31]. PSS experience additional specific challenges, such as financial pressures and adjustment to campus culture [8, 19] which position them as a highly distinctive group with unique mental health support needs and who may also face unique barriers or facilitators to engaging with mental health research. A distinct model or framework which addresses PSS‐specific needs for successful engagement is needed to support the growing field of campus mental health research [32], however, there is a current lack of understanding of existing models which have been used or recommended to guide PSS‐engaged mental health research.

Greenhalgh and team conducted a systematic review of existing frameworks utilized to guide patient engagement in research and developed a taxonomy for describing and understanding the diverse frameworks that exist [27]. Based on each framework's principles and practices for engaging patients in research, frameworks were grouped and classified into one of the five categories: (1) power‐focused (acknowledging and addressing power differences between researchers and patients/public), (2) priority‐focused (patients/public are involved in setting research focus areas), (3) study‐focused (patients/public are involved in designing and leading a target research project, typically tend to be clinical trials), (4) report‐focused (patients/public are involved in writing and reviewing research reports) and (5) partnership‐focused (patients and public members are partners in leading and disseminating research findings). Greenhalgh and colleagues emphasized the importance of reviewing and adapting existing frameworks to meet the needs of particular contexts or groups, and community‐specific models of engagement are being increasingly explored and developed for use in research [29, 33, 34]. Given the unique postsecondary context, and the increasing focus on PSS mental health research, there is a critical need to comprehensively review and adapt PSS‐specific engagement frameworks.

Therefore, guided by Greenhalgh's framework taxonomy [27], we aimed to undertake a scoping review to explore and characterize the frameworks that have been used to engage postsecondary students in campus mental health research, as well as to understand barriers and facilitators specific to the engagement of PSS in mental health research. Given the limited knowledge of existing frameworks to guide engagement with PSS in research, this study focused on identifying and mapping relevant engagement frameworks. A scoping review was identified as the most appropriate method to achieve these aims, allowing for a comprehensive overview of the literature [35].

We further aimed to collaborate with PSS to determine recommendations for student engagement and adapting, or developing, an engagement framework for PSS mental health research based on the findings of the review.

## 2 Methods

This scoping review was conducted following the five‐stage methodological framework by Arksey and O'Malley [36]. The Preferred Reporting Items for Systematic Review and Meta‐Analyses extension for Scoping Reviews (PRISMA‐ScR) [37] checklist was used to direct the reporting of this review. The protocol for this review is available upon request from the corresponding author [K.C.].

### 2.1 Stage 1: Identifying the Research Questions

#### 2.1.1 Literature Review Questions

1. What frameworks are used to guide the engagement of PSS in campus mental health research?
2. What are the facilitators and barriers to engaging PSS in campus mental health research?

#### 2.1.2 Guiding Question to Develop Recommendations Based on Review Findings

3. What recommendations do PSS have for implementing an engagement framework in PSS mental health research based on the findings of the scoping review?

### 2.2 Stage 2: Identifying Relevant Studies

To identify published academic articles, electronic database searches were initially conducted by a health sciences librarian on June 29, 2021. List of databases searched include Medline (Ovid), Embase (Ovid), CINAHL (EBSCO), Education Source (EBSCO) PsycInfo (Ovid), IBSS (ProQuest), ERIC (ProQuest) and Sociological Abstracts (ProQuest). The search strategy developed included use of terms focused on the following concepts: (1) postsecondary settings (e.g. college, university), (2) mental health and (3) engagement frameworks or models used to involve students in research (see Supplementary Information 1 for the search strategy used for each database). The definition of postsecondary (or tertiary) education can differ by countries and regions, as such we have utilized the World Bank's definition [38]: “all formal postsecondary education, including (i) public and private universities, (ii) colleges, (iii) technical training institutes, and (iv) vocational schools”. No limits or filters were used. Using the PRESS guidelines [39], a second health sciences librarian peer‐reviewed a draft of the OVID Medline search strategy before search translation. Following the initial deduplication of search results using EndNote [40], all searches were added to Covidence [41] where remaining duplicate searches were identified and removed. The search was updated on January 23rd, 2024, using the method outlined by Bramer and Bain [42].

A grey literature search was conducted following the guidelines outlined in the Grey Matters tool in 2021 and updated in January 2024. No relevant records were identified since most grey literature documents (e.g., reports, working papers) outlined practices and policies based on research findings without reference to the research methods and design (e.g., the type of engagement framework used); therefore, they did not meet this review's inclusion criteria outlined next.

### 2.3 Stage 3: Study Selection

Study selection was completed in two phases: initial title and abstract screening followed by full‐text screening of short‐listed abstracts, completed independently by two reviewers. Any conflicts for selecting studies during the two screening phases were reviewed and discussed by the research team, including the principal investigator (K.C.), before a final decision was made. Inclusion criteria for selecting studies were (1) written in English language, (2) based at a postsecondary setting (e.g. college, university), (3) describes PSS mental health research and (4) lists and/or describes the engagement framework used to involve students in the research process. Select document types, including conference abstracts, protocol papers, dissertations and editorials, were excluded.

### 2.4 Stage 4: Charting the Data

Two reviewers independently piloted data extraction of five articles to perform reliability comparison and review extraction results with the principal investigator (K.C.). Once reliability was established, data from the remaining documents was extracted by a single reviewer and entered into Microsoft Excel. The type of data extracted include year and country of publication, research questions, aims/objectives, study design used, population of interest, sample size, researcher composition (to identify number of students involved in research), methods, name and description of engagement framework used and facilitators and barriers to engaging students and/or using frameworks to engage students.

### 2.5 Stage 5: Collating, Summarizing and Reporting the Results

To address research Question 1, a list of engagement frameworks used across documents was generated. Two reviewers grouped the identified frameworks using Greenhalgh's taxonomy of 5 frameworks on supporting and evaluating patient and public involvement in research [27]: power‐focused, priority‐setting, study‐focused, report‐focused and partnership‐focused. We reviewed principles and characteristics associated with the framework as well as practices implemented as part of the framework. Where the study frameworks included characteristics or features which could be placed in more than one category, we categorized them according to best fit. Specifically, they were categorized based on how the study team described their understooding and implementation of the principles of the framework within their research. To address research Question 2, we extracted and compiled a list of barriers and facilitators reported while engaging PSS in mental health research. Barriers and facilitators have been classified into two categories previously used for youth engagement in mental health research: relational and process [22]. Best practices of patient engagement have been referred to as being presented within ‘frameworks’ and ‘models’ [26]. We adopted the term ‘framework’ for this review, as used in Greenhalgh et al.'s taxonomy [27].

### 2.6 PSS Engagement in This Review

A key recommendation while conducting a scoping review is to consult with research partners [36, 43], as it enhances the quality of this review and facilitates knowledge dissemination [43]. Thus, the study team engaged with an established campus‐based student mental health research advisory group, inclusive of the three listed student co‐authors [CL, OL, SP] to lead this review. The advisory group brings a diverse range of expertise relevant to student mental health and well‐being, including research, advocacy and lived experience expertise. During Stages 4 and 5 described above, the study team met with the student research advisory group to conduct two consultations on early findings from the review. Ahead of both meetings, student advisors extensively reviewed the shared findings and developed feedback to share with the study team. At the first meeting, students reviewed findings summarized from all documents, which included descriptions of engagement frameworks used, barriers and facilitators listed, and the initial categorization of frameworks using Greenhalgh's taxonomy [27], to assist with effectively synthesizing and reporting the results of this review. Student advisors provided feedback regarding revising the description of select frameworks, specifically when the distinction between certain frameworks was not clear (e.g., participatory action research and codesign). Students also advised reclassifying select frameworks within Greenhalgh's taxonomy [27]; they specifically recommended classifying frameworks based on how the authors described them, which resulted in the same framework reported across multiple documents being classified under different categories (e.g., participatory action research is classified under both study‐focused and partnership‐focused frameworks, as described below). All tables reporting study results were updated based on students' feedback. During the second meeting, the study team presented updated tables reflecting student recommendations for approval; the three student co‐authors also self‐selected to continue supporting this review, including preparing the manuscript during this meeting. Students then developed recommendations for implementing an engagement framework specifically for PSS mental health research. After finalizing the results, the student co‐authors identified key findings to be explored in the discussion section and outlined discussion points to be further developed in the reporting stage. Additionally, the three listed student co‐authors reviewed and revised this manuscript.

## 3 Results

### 3.1 Characteristics of Included Documents

**Figure 1:**
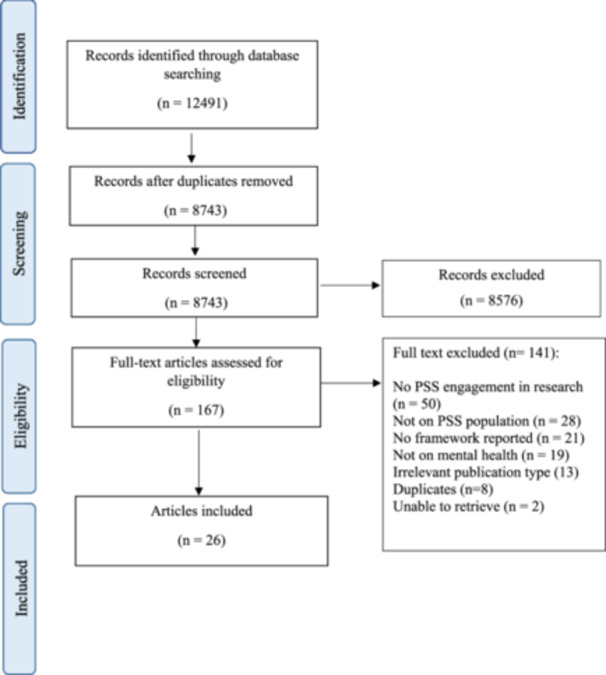
PRISMA flow chart of article selection.

Figure 1 shows the PRISMA flow diagram, which outlines the process of article selection. The total number of records identified was 12,491. Following the deduplication of records, the title and abstract of 8743 records were screened. 167 full‐text articles were screened to assess eligibility; of those, 26 articles were included in this review.

The characteristics of the included articles (*n* = 26) are presented in Table 1. The research methodology used across studies included qualitative (*n* = 12; 46%), quantitative (*n* = 7; 27%) and mixed‐methods (*n* = 7; 27%). Most studies (*n* = 18) used an observational design, while the remaining eight studies used an experimental design for evaluating interventions developed to support PSS mental health. The majority of the studies (73%) included samples of university students (*n* = 19); five studies included college students, and two studies included both college and university students.

**Table 1:** Characteristics of included journal articles.

Citation	Country of origin	Aims	Study Design	Population description and sample size	Main findings
Alsopp et al. [44]	United States	Explores university students perception regarding the influence of social factors on student mental health and well‐being	Qualitative – interviews	University students (*n* = 33)	Students reported multiple factors, such as academic competition and social isolation, which adversely impacted the mental health of university students.
Armstrong et al. [45]	United Kingdom	Describes university students' perspectives regarding use of mental health labels	Qualitative – interviews	University students (*n* = not reported)	Students attributed the use of mental health labels positively as it assisted them in describing their mental health needs and preferences to the university.
Benjamin et al. [46]	United States	Explores PhD students' experiences of well‐being during their transition into graduate school.	Qualitative – interviews	Graduate university students (*n* = 29)	Factors affecting the well‐being of doctoral students were identified and classified into three themes: being able to (1) manage relationships, (2) maintain health and (3) find purpose and place.
Blee et al. [47]	Australia	Investigates university students' perceptions regarding mental health concerns existing on‐campus to inform the development of a health promotion intervention for students.	Qualitative – focus group	Undergraduate and graduate university students (*n* = 13)	To develop and successfully implement health promotion interventions on campus, students described the need for (1) minimizing harm messaging, (2) recognizing the impact of untreated mental health problems and prioritizing early intervention and (3) addressing stigma and concerns about confidentiality.
Cage et al. [48]	United Kingdom	Examines barriers preventing university students from accessing support for mental health concerns.	Quantitative – surveys	Undergraduate and graduate university students (*n* = 376)	Barriers preventing students from accessing mental health supports were identified as perceived public stigma, self‐stigma, as well as existing mental health concerns among students.
Darker et al. [49]	Ireland	Describes students' involvement in the codesign and evaluation of an electronic health tool designed to support student mental health.	Mixed methods – quantitative (surveys) and qualitative (focus group)	First year undergraduate university students Quantitative: Initial survey: *n* = 120 Final survey: *n* = 196 Qualitative: First year students: *n* = 14 Peer supporters (undergraduate students): *n* = 6	Students provided feedback on the pilot version of the tool, such as adding more interactive components and revising language. The feedback was integrated into the final version by the research team, which included two students.
De Moissac et al. [50]	Canada	Explores students' perceptions of current mental health issues, existing resources available on campus, and recommended strategies to improve student mental well‐being on campus.	Qualitative – group discussion	University students (*n* = 73)	Students expressed concerns over increasing prevalence of mental health issues among postsecondary students. To improve student well‐being on campus, students recommended to: (1) provide students' access to counselling services; (2) support them with balancing academic and social commitments; and (3) integrate their voices in decisions that concern them.
Dooley et al. [51]	United Kingdom	Describes the impact of involving a student advisory group during the design and conduct of a national research project on student well‐being	Quantitative – surveys	Undergraduate and post‐graduate university students (*n* = 142) Researchers (*n* = 6)	Both research staff and student advisors attributed the involvement of student advisors in the project as largely positive. Recommendations by both groups for improving engagement work in future are to recruit a diverse group of student advisors for representation and ensure clear communication is maintained between the research team and the student advisory group.
Ehlinger et al. [52]	United States	Explores alcohol use among transgender and gender nonconforming (TGNC) college students.	Qualitative – focus group and interviews	College students (*n* = 16)	Three themes were identified regarding: (1) experiences unique to TGNC students on campus; (2) students' ongoing coping strategies for gender‐based discrimination and stressors and (3) reflection on existing programmes and suggestions for future intervention targeting gender‐based discrimination and alcohol use among TGNC students.
Farrer et al. [53]	Australia	Describes the development and evaluation of an online programme designed to support student mental health.	Mixed methods – quantitative (surveys) and qualitative (focus groups)	University students Quantitative: (*n* = 611) Qualitative: (*n* = 19)	Students co‐developed the online programme to include multiple features such as information on mental health, screening and self‐help tools. Students provided positive feedback regarding the participatory design process used.
Hickie et al. [54]	Australia	Describes the codesign, implementation and evaluation of an online platform to support student and youth mental health.	Mixed methods –quantitative (surveys) and qualitative (interviews)	First year university students Qualitative: (*n* = 16)	Students participated in codesign workshops to develop an online platform to support the wellbeing of university students. Features of the platform included an initial assessment for students to complete, following which they are provided with a personalized plan for tracking and achieving their identified goals.
Hotez et al. [55]	United States	Examines college students' mental health experiences during the COVID‐19 pandemic.	Mixed methods – quantitative (surveys) and qualitative (open‐ended questions asked in the survey)	College students Quantitative and qualitative: Wave 1 (*n* = 128) Wave 2 (*n* = 240)	A significant proportion of students reported experiencing mental health challenges during the pandemic across two waves – June and December 2020. Students reported higher levels of depression and anxiety in December 2020 in comparison to students who participated in June 2020.
Jack et al. [56]	United Kingdom	Describes the impact of migration on refugee students and the role of higher education institutions in supporting their needs.	Qualitative – interviews	Undergraduate and graduate university students (*n* = 9)	Refugee students reported barriers to seeking support for their well‐being at the university, including lack of awareness regarding the services available and stigma related to their cultural identity. Students recommended improving services for refugee students, including training therapists to develop cultural competence so they can better support refugee students.
Lira et al. [57]	Chile	Describes students evaluation of a mobile‐based intervention designed to address depression and anxiety concerns among students	Qualitative – workshops	University students (*n* = 13)	Students evaluated the application as being easy to use, with most finding it useful for tracking daily activities such as sleep and exercise. Students recommended improving the application by adding information regarding topics related to university life and mental health support available for students.
Lusk and Fearfull [58]	United Kingdom	Describes the implementation and evaluation of a programme to support university students' mental wellbeing.	Quantitative –surveys	University students (*n* = not reported)	As an alternative to the traditional counselling model, the support advisor model was implemented, offering students short‐term, individual and group support on campus. The strengths of implementing this model were that it increased capacity to accommodate more students and reduced wait times compared to the counselling model.
Mousavi et al. [59]	United States	Describes initiatives developed and led by the university's department to support graduate students mental health.	Quantitative – surveys	University graduate students (*n* = not specified) (as listed response rates of completion)	Students and university staff collaborated to develop a mental health survey for graduate students to complete. Survey results found that graduate students experienced high levels of stress yet did not seek help for mental health.
Murphy‐Shigematsu et al. [60]	United States	Identifies stressors experienced by Asian American students at a university setting.	Qualitative – focus groups	University students (*n* = 47)	As part of the evaluation of the engagement process, students reported gaining new knowledge and skills related to mental health and recommended assigning specific roles and responsibilities to students for engagement work in future.
Nicolaidou et al. [61]	Cyprus	Describes the design, feasibility, acceptance and usability of a mobile‐based application designed to serve as a mental health prevention intervention for students.	Quantitative – survey	Undergraduate university students (*n* = 74)	Students rated the application as significantly useful, which reflected high feasibility of the application. Students also expressed strong interest in continuing to use the application.
Olson et al. [62]	United States	Explores the impact of COVID‐19 lockdown on college students' mental health.	Quantitative – surveys	Undergraduate and graduate university students (*n* = 116)	Students reported poor mental health outcomes, attributing to significant stress due to remote learning. Students also described engaging in maladaptive coping strategies during the lockdown.
Park et al. [63]	United States	Describes mental health concerns among college students during the COVID‐19 pandemic	Quantitative – surveys	College students (*n* = 616)	Students reported high levels of anxiety, depression and suicidal ideation, as well as experiences of loneliness during the pandemic. Students also described their coping strategies, such as watching television.
Skoy and Werremeyer [64]	United States	Investigates the experiences of college students with mental health concerns.	Qualitative – interviews and focus group	College students (*n* = 12)	Based on students' reports of ongoing mental health concerns and its impact on their college experience, four themes were identified: (1) insights into campus mental health services, (2) increasing awareness and educating college community, (3) the need for support and (4) barriers to improving mental health.
VanHeerwaarden et al. [65]	Canada	Describes the implementation of participatory design research to develop a mobile‐based application to support student mental health.	Qualitative – interview and focus group	University and college students (*n* = 41)	Students shared their recommendations to improve the application, such as fixing glitches and improving layout, during the Codesign workshops. As part of the workshop evaluations, students provided positive feedback, including that they enjoyed the Codesign process and felt safe sharing their thoughts and opinions.
Vereschagin et al. [66]	Canada	Describes the co‐development of a mobile‐based application to support student wellbeing	Mixed methods – Quantitative (surveys) and qualitative – (focus groups)	University students Surveys (*n* = 101) Focus group (*n* = 14)	Students collaborated with the research team in three phases: conceptualization and initial application design, iterative user testing and final application design. Student feedback was integrated into developing and finalizing the design and content of the application.
Wada et al. [67]	Canada	Explores students' perspectives regarding existing stigma towards mental illness on campus.	Qualitative – interviews and focus group	Undergraduate and graduate university students (*n* = 24)	Based on students' descriptions of stigma about mental health on campus, three themes were identified: 1) academic pressure and lack of understanding of illness identified as sources of stigma, 2) mental illness stigma can negatively impact a student's life and, 3) the need to reduce mental illness stigma through increasing awareness on campus.
Werremeyer et al. [68]	United States	Investigates the use and impact of photovoice as an intervention for college students with mental health illnesses.	Mixed methods – quantitative (surveys and journal) and qualitative (interviews)	College students (*n* = 10)	Participants in the photovoice intervention group had significant reduction in anxiety scores as compared to the control group. No improvement was seen in the depression and medication adherence scores in both groups.
Wiljer et al. [69]	Canada	Describes the codesign methods used to develop a mobile‐based application for student mental health.	Mixed methods – quantitative (surveys) and qualitative (interviews)	University and college students (*n* = 10)	Students were engaged in the Codesign workshops held for initial development and evaluation of the application. As part of the evaluation of the engagement process, students reported having gained new knowledge and skills related to mental health.

Question 1 Frameworks used for engaging students in campus mental health research

The frameworks reported in the included documents are described in Table 2. We found a predominant use of frameworks falling under the umbrella of participatory research [70] and ‘co'approaches (e.g., codesign, coproduction) [71] used for engaging patients and/or public in research. In classifying the frameworks using Greenhalgh's taxonomy [27], we found a lack of standardization around the description of frameworks. Authors often reported using a specific engagement framework, but studies operationalized and utilized a single framework in different ways. For example, documents that reported utilizing participatory action research (PAR) described this framework differently. One study described having used PAR to improve the process and quality of their research project (i.e., study‐focused framework) [55] while another employed it to establish and enhance partnerships with students (i.e., partnership‐focused framework) [60]. As a result, studies using PAR (and other frameworks) were sorted across different framework categories depending on how it was described in the document. Table 3 identifies the categorizations of the included frameworks according to Greenhalgh's taxonomy [27].

**Table 2:** List and description of the frameworks and their subcategories, where applicable, that were used to engage PSS in campus mental health research.

Framework	Subcategory	Description
Participatory research	1. Participatory Action Research (PAR) [47, 48, 55, 58, 60, 67] 2. Participatory Design Research (PDR) [53, 57, 61, 65] 3. Community‐based participatory research (CPBR) [52] 4. Photovoice (PV) [56, 68] 5. Participatory course‐based undergraduate research experience [63]	1. The primary objective of participatory action research (PAR) is to initiate change within the particular system being researched. Implementation of PAR involves collaboration between researchers and identified research partners (e.g., students) in identifying and addressing target issues within the system (e.g., mental health) [60, 67]. 2. Participatory Design Research (PDR) involves the engaging research partners in developing, prototyping and testing products or services (e.g., mobile apps) [53]. 3. Community‐based participatory research (CPBR) involves an active partnership where power sharing is prioritized between researchers and communities to lead research initiatives relevant to the community [52]. 4. Photovoice is a participatory action research method, where partners engage in research through capturing and providing photographs related to a given research topic. Photographs are analyzed during individual as well as group discussion among all partners involved in the project [56, 68]. 5. As part of the participatory course‐based undergraduate research experience, students selected their research topic of interest, following which they designed the survey, conducted data analysis and shared the findings with the student body and other university members. Students partnered with a faculty member to lead their project during and after the completion of the course [63].
Co‐approaches	1. Codesign [49, 65, 69] 2. Coproduction [44, 45] 3. Co‐development [66]	1. Following the Codesign framework, research initiatives are jointly led by researchers and members belonging from the target research population, subject matter experts and knowledge users [47, 58]. While use of PAR is intended to initiate a social change [60], Codesign is typically employed for the research and development of products that meet the needs of end‐users [69]. 2. Coproduction involves collaboration among researchers and community members specifically those belonging from marginalized communities [44]– for varying research purposes, such as determining the research design and presenting findings [45]. 3. Following the co‐development framework, researchers and identified partners collaborate to design a target product across three phases: conceptualization and initial design, user testing and final design [66].
World Café [50]	NA	In a world café format, a group discussion is organized between researchers and identified partners to brainstorm and share ideas regarding a selected research topic; researchers typically record the discussion for analysis [50].
Photo‐elicitation [46, 62]	NA	Photo‐elicitation involves similar methods as PV, given that partners are engaged in research through capturing photographs. Unlike PV, photo‐elicitation does not involve partner input during data analysis [46, 62].
Custom‐designed frameworks	1. Chem. Dept. mental health improvement initiative at the University of Minnesota [59] 2. Nurture‐U Student Advisory Group [51]	1. The chemistry department at the University of Minnesota established a mental health‐focused initiative, which included collaboration between students and university leadership in developing a research survey to assess students' mental health concerns [59]. 2. A student advisory group (SAG) was established to inform the research design and support operations of the Nurture U project on student well‐being. Student advisors were engaged across multiple activities including developing recruitment strategies for collecting survey data, testing tools being used for data collection (I.e., REDCap) and co‐designing a toolkit on student wellbeing [51].

**Table 3:** Categorization of framework using Greenhalgh et al.'s taxonomy.

Greenhalgh et al.'s Taxonomy [27]	Frameworks used to engage PSS in mental health research
**Power‐focused frameworks** Identifying and challenging power differences between researchers and individuals engaged in research (i.e., patients and/or public members)	1. Community‐based participatory research [52] 2. Photo‐elicitation [46] 3. Coproduction and critical pedagogy [44]
**Priority‐setting frameworks** Engaging with patients and public members to identify research priorities.	None available.
**Study‐focused frameworks** Establishing and facilitating patient and public engagement throughout the research process, from drafting the initial application to sharing findings, to enhance the quality and impact of the research study.	1. Participatory action research [48, 55] 2. Photovoice [56, 64, 68] 3. Codesign [49, 54] 4. Photo‐elicitation [62] 5. Participatory design research [53, 57, 61] 6. Chemistry department mental health improvement initiative [59] 7. World café [50] 8. Co‐development [66]
**Report‐focused frameworks** Guidelines on reporting patient and/or public members’ engagement during the research process.	None available.
**Partnership‐focused frameworks** Focuses on optimizing partnership between research and patients and public members through adopting practices to facilitate effective engagement (e.g., establishing consistent and clear communication, providing training to both researchers and patient/public members, etc.) and measuring the partnership's impact.	1. Participatory action research [47, 58, 60, 67] 2. Codesign [69] 3. Participatory design research [65] 4. Coproduction [45] 5. Nurture‐U Student Advisory Group [51] 6. Participatory course‐based undergraduate research [63]

### 3.2 Power‐Focused Frameworks

Central to this category of frameworks is identifying and challenging power differences, with the goal of promoting power sharing between researchers and individuals with lived experience who are engaged in research [27]. Three studies [44, 46, 52] reported using frameworks which fell into this category. Ehlinger et al. [52] reported using community‐based participatory research (CBPR), which they described as aiming to minimize researcher bias through establishing a meaningful partnership between researchers and community members (e.g., transgender and gender nonconforming college students in their study) to cocreate knowledge and change within the community. The other two frameworks classified in this category are photo‐elicitation [46] and coproduction design with principles of critical pedagogy integrated [44]. Benjamin et al. [46] described using photo‐elicitation, asking participants to self‐direct data collection through photo capture with the aim of equalizing the role between researcher and participant. Meanwhile, Alsopp et al. [44] described using the coproduction framework while applying principles of critical pedagogy to colead the research study with students, arguing that using a coproduction framework itself is not sufficient to address power imbalances among researchers and students.

### 3.3 Priority‐Setting Frameworks

This framework category was defined as those describing a process for collaboration amongst researchers and individuals to identify and establish priorities for research (i.e. establishing topics that should be pursued in research) [27]. None of the included documents reported using frameworks or models which aligned with this category.

### 3.4 Study‐Focused Frameworks

Most of the studies (*n* = 14) reported using frameworks which aligned with the study‐focused category, which are frameworks focused on how patients or persons with lived experience can be involved throughout the different stages and procedures of conducting a study [27]. Characteristics of these frameworks broadly include examining and understanding the research context; ensuring proper advanced planning and support for different study stages; avoiding tokenism in engagement efforts; supporting inclusivity; building and maintaining trusting relationships with patient partners, etc [27]. This category included studies where PSS were engaged in research targeting product or service design, particularly in advanced planning and establishing ongoing relationships with PSS in research. Specifically, this included studies that employed codesign [49, 54], co‐development [66] and participatory design research [53, 57, 61] where PSS were engaged in research and development of online applications and tools to support student mental health. Two studies that engaged with students to enhance student mental health services have also been classified in this category (engagement framework designed for the chemistry department mental health initiative [59] and World Café [50]).

Studies included in this category used photovoice [56, 64, 68] and photo‐elicitation [62], where these frameworks have been described as strengths of the study design, aligning them with the intent of study‐focused frameworks to enhance the quality of research. Two studies [48, 55] that described employing PAR have been categorized here since they described the involvement of students throughout the research process. For example, Hotez et al. [55] described engaging with students in the design, dissemination and analysis of the research survey investigating the impact of the COVID‐19 pandemic on college students' mental health.

### 3.5 Report‐Focused Frameworks

These frameworks include guidelines on reporting patient and/or public engagement in research [27], for example, the GRIPP‐2 checklist for appraising studies on their reporting of different items related to patient engagement in the research process [72]. We found no studies in our search which reported using a report‐focused framework.

### 3.6 Partnership‐Focused Frameworks

A total of 9 studies reported using partnership‐focused frameworks, defined by Greenhalgh [27] as those optimizing individual engagement in research. Greenhalgh [27] identified common themes of this framework category as: developing power‐sharing arrangements, establishing meaningful and consistent communication, providing training (to both researchers and partners), and evaluating engagement processes and impact. Four studies reporting use of PAR [47, 58, 60, 67] or a type of PAR approach (e.g. participatory course‐based undergraduate research) [63], which, as described, aligned most closely with this category due to their reported processes, such as implementing training. For example, Murphy‐Shigematsu et al. [60] reported providing extensive training to students spanning across eight sessions where students learned about study design, data collection and analysis. These PAR studies also emphasized the active role of students as “active research members” [67] or “researchers” [47]. Murphy‐Shigematsu et al. [60] defined student research partners as “consultants”. This vocabulary reflects an attempt towards establishing balance and power between researchers and students in leading research, which is central to both the principles of power and partnership‐focused framework [27]. However, we classified them under partnership‐focused, as researchers described using elements beyond power‐sharing, such as training and/or evaluation, to facilitate student engagement in research.

Three studies that reported using co‐development [45], codesign [69] and participatory design research [65] were categorized within this group. Two studies [65, 69] are from the same research group, describing engagement activities and outcomes through engaging PSS in co‐designing a mobile application to support PSS in navigating and accessing mental health services. PSS in these studies were trained in research [65], as well as provided opportunities to lead workshops and activities for supporting the crowdsourcing of resources [69]. VanHeerwaarden et al. [65] also described conducting an evaluation of the workshops held where students provided positive feedback that they felt safe sharing their thoughts, as well as recommendation for allocating adequate time for these workshops. These components of providing training and leadership opportunities to students and evaluating the engagement processes aligns with the principles of partnership‐focused research. A single custom‐designed framework (e.g., the Nurture‐U student advisory group) [51] has been categorized as partnership‐focused due to its practices of power‐sharing with PSS throughout their research project and adoption of the ‘patient engagement guidance tool’ [73] specifically for evaluating the quality of student engagement.

Question 2 **Facilitators and barriers to engaging students in mental health research**

Seven studies reported facilitators and/or barriers (see Table 4) to engaging PSS in mental health research, with most suggestions coming from studies which reported implementing PAR or codesign frameworks.

**Table 4:** List of barriers and facilitators to engaging PSS in campus mental health research.

	Facilitators	Barriers
**Relational**	Creating a comfortable and safe environment [50] Listening to students without expectations [60] Mitigate effects of power imbalance between researchers and students (e.g. through establishing mutual respect and clear communication) [51, 65]	Conflicting feedback from students may impact decision‐making [51, 65]
**Process**	Using diverse and multiple approaches to collect feedback from students (e.g., paper‐based approaches for written feedback [69]; online white board where students can post feedback anonymously [51]) Facilitating small group discussions [54, 65] and/or individual methods for providing feedback (I.e., using padlet [51]) to foster participant confidence. Establishing student roles and responsibilities [69] Providing training to students (e.g. on topics related to research process) [69]	Lack of continuous funding [53] Time commitment needed to implement an engagement framework [52, 53] Lack of resources may impact implementation of student feedback [53]

Facilitators included principles such as a focus on a safe environment, respect and communication [50], as well as practical steps such as using small groups and paper‐based approaches to encourage engagement [51, 54, 65]. Clearly establishing roles and providing training were also highlighted as facilitators [69].

Barriers described included issues around resource and time constraints [52, 54], including challenges in scheduling students given their busy academic calendars and competing priorities. Further, within certain frameworks it can be challenging to establish or to know how to manage conflicting perspectives from different student partners [51, 65].

Question 3 **Recommendations for a student‐specific model of engagement in student mental health research**

During consultations with our student advisory team, they expressed a need to develop a student‐specific model of engagement in PSS mental health research, as none of the frameworks discussed met the unique needs of the PSS population. As indicated by one student: “I do think all of the frameworks are necessary but each on its own is not sufficient to encapsulate the best practices for student engagement in [student mental health research]”. Student recommendations build upon existing frameworks to meet the identified gaps in engagement models for PSS mental health research. Recommendations can be divided into four overarching categories: (1) account for the postsecondary context, (2) prioritize clear communication throughout the engagement process, (3) develop a shared understanding of responsibilities and (4) focus on accountability and meaningful engagement. See Table 5 for a summary of the recommendations given by students.

**Table 5:** Recommendations for a postsecondary student‐specific model of engagement in mental health research.

Recommendation	
Account for the postsecondary context	Tailor recruitment activities for diverse student groups and project members. Different recruitment strategies and best practices are needed to account for diversity within postsecondary settings. Strategies to ensure continuity and effective transitions among engaged students are needed to account for programme‐dictated turnover in certain PSS populations (i.e., students transitioning into first year and senior students graduating). Recognize the financial barriers many PSS face (i.e., increased cost of living, tuition), and include financial compensation, where possible.
Prioritize clear communication throughout the engagement process	Develop best practices to communicate what students need to know at the beginning of the project. This includes, but is not limited to, how decision making will happen, how to address power dynamics, and how to determine the scope of each partners' role. Develop strategies for continually engaging students, with particular focus directed towards engagement during high‐stress times of the year (i.e., exam season).
Develop a shared understanding of responsibilities	Develop standards for how project priorities and/or deliverables are identified, ensuring equal input from all project partners. Ensure that frameworks are tailored to the specific needs and roles of all partners involved in the research process. For example, separate principles for student engagement should be created for federal funding agencies, postsecondary institutions and independent researchers.
Focus on accountability and meaningful engagement	Develop principles to ensure that student engagement is genuine and focuses on building trust. Develop guidelines outlining timely and detailed follow‐up with students who have been engaged in the research process.

## 4 Discussion

This is the first review, to our knowledge, that identifies and maps engagement frameworks for PSS research. Using Greenhalgh and colleagues [27] taxonomy, our review identified five (of seven) frameworks that have been used to engage postsecondary students in campus mental health research: participatory action research, codesign, World Café, photoelicitation and two custom‐designed frameworks that did not fall into the other framework categories (i.e., chemistry department mental health initiative [59] and Nurture‐U student advisory group [51]).

Two key challenges were identified with existing models of postsecondary student engagement: underreporting of frameworks and lack of consistency in common language. These challenges highlight the need for a clear, actionable engagement model for PSS, which includes comprehensive descriptions of how different components can be implemented within a research study. First, existing engagement models and frameworks specific to the postsecondary context have yet to be comprehensively developed, leading to inconsistencies across the literature. Much of the existing literature included in this review lacked a clear description of the framework employed. This was particularly salient with participatory action frameworks; for example, Murphy‐Shigematsu et al. [60] and Hotez et al. [55] both reported utilizing PAR frameworks, yet described different steps for applying this model. Often, a specific framework would be referenced with minimal description of what the framework entailed or how it was specifically implemented, leading to challenges in synthesizing findings. The lack of clearly and consistently defined frameworks to guide the ‘how‐to’ of PSS engagement in research risks precluding research from being able to effectively adopt engagement best practices into ongoing work [74]. Further, inconsistent understanding and utilization of frameworks makes rigorous evaluation of PSS engagement practices, which is needed to understand the potential benefits of co‐leading research with PSS, methodologically challenging [75]. The second key challenge was lack of common language to describe engagement activities and the role of PSS in the research process. For example, when describing engagement activities, some authors described PSS as researchers or co‐researchers [47, 60], while others referred to PSS as research participants [64, 68]. Though it is possible that students who were referred to as “research participants” were effectively engaged in the research process, the lack of consistency in terminology challenges our understanding of the extent to which PSS were engaged in each study. This has also been identified in the wider literature on patient and public engagement in research as a key challenge [76]. Operationalization of consistent language to describe the different roles of PSS engaged in research activities will contribute to more robust engagement frameworks and shared understanding of how PSS perspectives are being integrated into studies across researcher and knowledge user groups.

### 4.1 Barriers and Facilitators of Student Engagement in PSS Mental Health Research

Given the lack of consistency in reporting the five framework types, it was challenging to comprehensively identify barriers and facilitators specific to each. We therefore summarized barriers and facilitators that were reported across the five engagement frameworks included in this review. Most of the identified barriers, such as lack of funding to conduct patient engagement, challenges with time commitments and lack of resources to actually implement feedback from patient partners, have been identified in the broader literature on patient engagement in general [20, 22, 77]. Further, facilitation strategies such as providing training to patient experts or clearly establishing roles and responsibilities have also been shown to support patient engagement in other areas [19, 21].

There are, however, certain unique characteristics of the PSS population that require a tailored engagement framework as well as supports to enhance student engagement in research. For example, students' personal commitments and timelines were noted as a potential barrier for engagement [53]. PSS's level of commitment fluctuates with the academic calendar and the nature of moving through an academic degree or diploma programme is such that students may complete their semesters or programmes before the conclusion of a study, thus creating challenges with continuous engagement throughout the typical lifespan and phases of a study. Processes described within engagement frameworks may need to incorporate alignment with the academic calendar to minimize burdens on PSS co‐researchers as well as ensure that student expertise is being integrated consistently across a study.

Attending to power differentials was also among the identified facilitators. Unattended differences in power between researchers and patient partners have previously been explored in the literature on patient engagement as a challenge to safe and authentic engagement [22, 25]. Specifically, prior research has spoken to the risk of a “co‐opted relationship”, wherein there is a focus on inducting and assimilating the patient partner into the research environment, rather than on how the patient partner may lead aspects of research and influence the status quo [77]. This type of relationship, which has been described as being similar to a supervisor‐student relationship [77] may be a greater risk for PSS, who are receiving training and education in the same setting where they are engaging as experts with lived experience. As mentioned in the previous section, challenges exist with vocabulary used to describe PSS partners and the boundaries between these roles may be further complicated as PSS inhabit roles as trainees and research assistants in studies. Strategies such as implicit bias training or exercising critical reflectivity to enhance researcher awareness of their role and power [78] may help to mitigate some of the harmful power imbalances that exist in community‐ or patient‐engaged research. Particular attention should be paid to the potentially dual role as expert partner and trainee that students may be experiencing in the campus setting. Many of the studies included in this review did not explicitly refer to power imbalances when discussing barriers and facilitators to PSS engagement, making this a priority consideration for the development or tailoring of an engagement framework.

### 4.2 Student Driven Recommendations for an Engagement Framework for PSS Mental Health Research

As described in Table 5, students identified a range of recommendations that built upon components of all identified frameworks and emphasize the critical importance of adapting existing tools to meet current needs.

A core principle encompassing all recommendations provided by students is the need for a comprehensive reporting system of all engagement strategies utilized. Notably, we found no implementation of report‐focused frameworks in our review. Focusing on strong reporting will aid in successful implementation of validated student engagement strategies and will help to promote utilization and refinement of best practices. Students engaged in our consultations consistently emphasized the importance of clear reporting to ensure that engagement practices are continually strengthened. Integration of, and adherence to, reporting guidelines are recommended to address the lack of detail and consistency that is currently found in the reporting of patient engagement across disciplines [79]. The GRIPP2 reporting checklist for patient and public involvement in research [72] elicits clear commentary on researchers' definition of engagement, frameworks and engagement methods used, barriers and facilitators and other aspects of the engagement process. The GRIPP2 [72] could potentially be adapted for use in PSS engagement, with tailoring based on student‐driven recommendations. For example, the definition of engagement item (Item 2A) might include a requirement for clear reporting on the role of students to delineate trainee research assistants from PSS engaged for their lived expertise. Another example might be adapting the item that requires commentary on contextual factors that influenced patient engagement (8E), to include sub‐items specific to the unique student‐researcher power differential present in PSS engagement.

## 5 Limitations

There are potential limitations to using a pre‐established framework for categorizing and differentiating types of frameworks. This practice was helpful for organizing and generating an understanding of the overall scope of the literature on this topic and summarizing key themes from the diverse frameworks used in PSS‐engaged research. However, nuances that exist between different models/frameworks organized into the same category may have been missed. Additionally, while search terms related to student engagement were developed to be as expansive and sensitive as possible, given the heterogeneity of language used to describe student or patient engagement in research, some relevant documents may not have been captured.

## 6 Conclusion and Future Directions

This scoping review highlights the need for the development of a comprehensive model of engagement for PSS in mental health research. Building on feedback from students, the model should include a clear definition of student engagement, encompassing both how student engagement is operationalized and what successful student engagement looks like. Additionally, that lack of consistency in current frameworks accentuates a need for the development of reporting standards on PSS engagement or tailoring of existing standards such as the GRIPP2 reporting guidelines [72]. Reporting standards would aid in garnering more consistent research on PSS engagement models and their associated efficacy, while also enhancing the ease with which researchers can implement these frameworks. Reporting standards should incorporate a clear description of the framework utilized, a specific identification of where and how in the research process students were engaged, and feedback from students and staff involved. A tailored framework for supporting the implementation and reporting of PSS engagement will enhance both the quality and transparency of student engagement practices in the growing field of PSS mental health research.

## Author Contributions

**Kristin Cleverley:** conceptualization, investigation, funding acquisition, writing–original draft, methodology, writing–review and editing, formal analysis, project administration, supervision, resources, validation. **Soha Salman:** writing–original draft, writing–review and editing, formal analysis, validation. **Julia Davies:** writing–review and editing, writing–original draft. **Lexi Ewing:** writing–original draft, writing–review and editing, formal analysis. **Emma McCann:** conceptualization, writing–review and editing, resources, writing–original draft. **Katherine Sainsbury:** writing–original draft, writing–review and editing, formal analysis. **Mikaela Gray:** conceptualization, investigation, writing–original draft, writing–review and editing, resources. **Carrie K. Y. Lau:** writing–review and editing, validation, writing–original draft. **Orly Lipsitz:** writing–original draft, validation, writing–review and editing. **Sapolnach Prompiengchai:** writing–original draft, writing–review and editing, validation.

## Conflicts of Interest

The authors declare no conflicts of interest.

## Supporting information

Supporting information.

## Data Availability

All data generated or analyzed during this study are included in this published article and/or its supporting information materials.
