# Supplementary material for: Frameworks Used to Engage Postsecondary Students in Campus Mental Health Research: A Scoping Review
Source: Health Expect. 2025 Mar 21;28(2):e70144. doi: 10.1111/hex.70144 (PMC11926649; doi:10.1111/hex.70144)
Supplement: Supplementary file 1 — Supporting information. [file HEX-28-e70144-s001.docx]

**Supplementary file 1 – Search strategy for each database**

## Updated Searches – January 23^rd^, 2024

### Ovid Medline

**Ovid MEDLINE: Epub Ahead of Print, In-Process & Other Non-Indexed Citations, Ovid MEDLINE® Daily and Ovid MEDLINE® <1946-Present>**

| # | Searches | Results |
| --- | --- | --- |
| 1 | Mental Health/ | 65009 |
| 2 | Mental Disorders/ | 179512 |
| 3 | ((mental* or psychiatric* or psychological*) adj3 (health or disorder* or illness* or ill or problem* or disease*)).tw,kf. | 421706 |
| 4 | ((mental* or psychiatric* or psychological*) adj3 (wellbeing or well-being)).tw,kf. | 31650 |
| 5 | wellness.tw,kf. | 15617 |
| 6 | or/1-5 [Mental Health] | 552596 |
| 7 | Community-Based Participatory Research/ | 5727 |
| 8 | ((collab* or inclusi* or include* or participa* or action or community based or peer or communit* or together or team*) adj3 research*).tw,kf. | 107407 |
| 9 | (co adj (lead or creat* or design* or research* or enquire* or produce*)).tw,kf. | 7627 |
| 10 | (colead or cocreat* or codesign* or coresearch* or coenquire* or coproduce*).tw,kf. | 1861 |
| 11 | (student* adj4 (partner* or creat* or produc* or collab* or design* or engage* or drive* or change agent* or knowledge user* or oriented or centered or centred or advisor*)).tw,kf. | 21296 |
| 12 | partnership approach*.tw,kf. | 408 |
| 13 | Decision Making, Shared/ | 2075 |
| 14 | shared decision making.tw,kf. | 15265 |
| 15 | or/7-14 [Participatory Action Research] | 152624 |
| 16 | Universities/ | 53772 |
| 17 | (post-secondary or postsecondary).tw,kf. | 2599 |
| 18 | universit*.tw,kf. | 485489 |
| 19 | college*.tw,kf. | 154776 |
| 20 | campus*.tw,kf. | 14298 |
| 21 | (higher education or higher learning).tw,kf. | 22149 |
| 22 | tertiary education.tw,kf. | 1786 |
| 23 | ((graduate or undergrad* or under-grad* or doctora* or phd or master* or postgraduate*) adj2 (studies or education or institution* or student* or program* or course* or cohort* or training)).tw,kf. | 70830 |
| 24 | ((vocational or trade*) adj2 (school* or training or student* or studies or education or institution* or program*)).tw,kf. | 5272 |
| 25 | or/16-24 [Higher education] | 698019 |
| 26 | 6 and 15 and 25 | 1636 |

### Ovid Embase

**Embase Classic+Embase <1947 to 2024 January 22>**

| # | Searches | Results |
| --- | --- | --- |
| 1 | mental health/ or community mental health/ or psychological well-being/ | 252400 |
| 2 | mental disease/ | 312641 |
| 3 | ((mental* or psychiatric* or psychological*) adj3 (health or disorder* or illness* or ill or problem* or disease*)).tw,kw. | 525427 |
| 4 | ((mental* or psychiatric* or psychological*) adj3 (wellbeing or well-being)).tw,kw. | 38642 |
| 5 | wellness.tw,kw. | 20913 |
| 6 | or/1-5 [Mental Health] | 806859 |
| 7 | action research/ or participatory action research/ | 1920 |
| 8 | participatory research/ | 7468 |
| 9 | shared decision making/ | 16232 |
| 10 | ((collab* or inclusi* or include* or participa* or action or community based or peer or communit* or together or team*) adj3 research*).tw,kw. | 144092 |
| 11 | (co adj (lead or creat* or design* or research* or enquire* or produce*)).tw,kw. | 9531 |
| 12 | (colead or cocreat* or codesign* or coresearch* or coenquire* or coproduce*).tw,kw. | 2139 |
| 13 | (student* adj4 (partner* or creat* or produc* or collab* or design* or engage* or drive* or change agent* or knowledge user* or oriented or centered or centred or advisor*)).tw,kw. | 26881 |
| 14 | partnership approach*.tw,kw. | 547 |
| 15 | shared decision making.tw,kw. | 21450 |
| 16 | or/7-15 [Participatory Action Research] | 208279 |
| 17 | university/ | 132908 |
| 18 | college/ | 113588 |
| 19 | community college/ | 376 |
| 20 | vocational education/ | 12757 |
| 21 | student/ or college student/ or graduate student/ or phd student/ or postgraduate student/ or undergraduate student/ or university student/ | 190564 |
| 22 | (post-secondary or postsecondary).tw,kw. | 3095 |
| 23 | universit*.tw,kw. | 854253 |
| 24 | college*.tw,kw. | 294379 |
| 25 | campus*.tw,kw. | 19341 |
| 26 | (higher education or higher learning).tw,kw. | 26424 |
| 27 | ((graduate or undergrad* or under-grad* or doctora* or phd or master* or postgraduate*) adj2 (studies or education or institution* or student* or program* or course* or cohort* or training)).tw,kw. | 82288 |
| 28 | ((vocational or trade*) adj2 (school* or training or student* or studies or education or institution* or program*)).tw,kw. | 6940 |
| 29 | or/17-28 [Higher Education] | 1397809 |
| 30 | 6 and 16 and 29 | 2820 |

### Ovid PsycInfo

**APA PsycInfo <1806 to January Week 3 2024>**

| # | Searches | Results |
| --- | --- | --- |
| 1 | mental health/ | 93898 |
| 2 | mental disorders/ or chronic mental illness/ or serious mental illness/ | 101664 |
| 3 | well being/ | 62638 |
| 4 | ((mental* or psychiatric* or psychological*) adj3 (health or disorder* or illness* or ill or problem* or disease*)).tw. | 427950 |
| 5 | ((mental* or psychiatric* or psychological*) adj3 (wellbeing or well-being)).tw. | 31268 |
| 6 | wellness.tw. | 10482 |
| 7 | or/1-6 [Mental Health] | 513257 |
| 8 | action research/ | 4392 |
| 9 | ((collab* or inclusi* or include* or participa* or action or community based or peer or communit* or together or team*) adj3 research*).tw. | 87002 |
| 10 | (co adj (lead or creat* or design* or research* or enquire* or produce*)).tw. | 6581 |
| 11 | (colead or cocreat* or codesign* or coresearch* or coenquire* or coproduce*).tw. | 1260 |
| 12 | (student* adj4 (partner* or creat* or produc* or collab* or design* or engage* or drive* or change agent* or knowledge user* or oriented or centered or centred or advisor*)).tw. | 49826 |
| 13 | partnership approach*.tw. | 293 |
| 14 | shared decision making.tw. | 4004 |
| 15 | or/8-14 [Participatory Action Research] | 144249 |
| 16 | colleges/ or community colleges/ | 19957 |
| 17 | higher education/ or graduate education/ or postgraduate training/ or undergraduate education/ | 33254 |
| 18 | graduate schools/ | 801 |
| 19 | campuses/ | 2867 |
| 20 | college students/ or community college students/ or junior college students/ or graduate students/ or postgraduate students/ | 109734 |
| 21 | vocational education/ or vocational school students/ | 3685 |
| 22 | (post-secondary or postsecondary).tw. | 9209 |
| 23 | universit*.tw. | 193273 |
| 24 | college*.tw. | 246830 |
| 25 | campus*.tw. | 20661 |
| 26 | (higher education or higher learning).tw. | 39752 |
| 27 | tertiary education.tw. | 1084 |
| 28 | ((graduate or undergrad* or under-grad* or doctora* or phd or master* or postgraduate*) adj2 (studies or education or institution* or student* or program* or course* or cohort* or training)).tw. | 82061 |
| 29 | ((vocational or trade*) adj2 (school* or training or student* or studies or education or institution* or program*)).tw. | 8690 |
| 30 | or/16-29 [Higher education] | 482315 |
| 31 | 7 and 15 and 30 | 2333 |

### Ebsco CINAHL Plus with Full Text

| **#** | **Query** | **Results** |
| --- | --- | --- |
| S30 | S8 AND S17 AND S29 | 1,435 |
| S29 | S18 OR S19 OR S20 OR S21 OR S22 OR S23 OR S24 OR S25 OR S26 OR S27 OR S28 | 319,180 |
| S28 | TI ( ((vocational or trade*) N2 (school* or training or student* or studies or education or institution* or program*)) ) OR AB ( ((vocational or trade*) N2 (school* or training or student* or studies or education or institution* or program*)) ) | 3,270 |
| S27 | TI ( ((graduate or undergrad* or under-grad* or doctora* or phd or master* or postgraduate*) N2 (studies or education or institution* or student* or program* or course* or cohort* or training)) ) OR AB ( ((graduate or undergrad* or under-grad* or doctora* or phd or master* or postgraduate*) N2 (studies or education or institution* or student* or program* or course* or cohort* or training)) ) | 41,934 |
| S26 | TI tertiary education OR AB tertiary education | 825 |
| S25 | TI ( (higher education or higher learning) ) OR AB ( (higher education or higher learning) ) | 12,747 |
| S24 | TI campus* OR AB campus* | 8,916 |
| S23 | TI college* OR AB college* | 84,981 |
| S22 | TI universit* OR AB universit* | 178,551 |
| S21 | TI ( (post-secondary or postsecondary) ) OR AB ( (post-secondary or postsecondary) ) | 1,951 |
| S20 | (MH "Students, College") OR (MH "Students, Graduate") OR (MH "Students, Undergraduate") | 42,567 |
| S19 | (MH "Education, Graduate") OR (MH "Education, Doctoral") OR (MH "Education, Post-Doctoral") | 5,842 |
| S18 | (MH "Colleges and Universities") OR (MH "Community Colleges") | 31,019 |
| S17 | S9 OR S10 OR S11 OR S12 OR S13 OR S14 OR S15 OR S16 | 98,992 |
| S16 | TI shared decision making OR AB shared decision making | 7,769 |
| S15 | TI partnership approach* OR AB partnership approach* | 400 |
| S14 | TI ( (student* N4 (partner* or creat* or produc* or collab* or design* or engage* or drive* or change agent* or knowledge user* or oriented or centered or centred or advisor*)) ) OR AB ( (student* N4 (partner* or creat* or produc* or collab* or design* or engage* or drive* or change agent* or knowledge user* or oriented or centered or centred or advisor*)) ) | 17,260 |
| S13 | TI ( (colead or cocreat* or codesign* or coresearch* or coenquire* or coproduce*) ) OR AB ( (colead or cocreat* or codesign* or coresearch* or coenquire* or coproduce*) ) | 783 |
| S12 | TI ( (co N0 (lead or creat* or design* or research* or enquire* or produce*)) ) OR AB ( (co N0 (lead or creat* or design* or research* or enquire* or produce*)) ) | 4,485 |
| S11 | TI ( ((collab* or inclusi* or include* or participa* or action or community based or peer or communit* or together or team*) N3 research*) ) OR AB ( ((collab* or inclusi* or include* or participa* or action or community based or peer or communit* or together or team*) N3 research*) ) | 65,953 |
| S10 | (MH "Decision Making, Shared") | 3,882 |
| S9 | (MH "Action Research") | 7,614 |
| S8 | S1 OR S2 OR S3 OR S4 OR S5 OR S6 OR S7 | 308,892 |
| S7 | TI wellness OR AB wellness | 14,377 |
| S6 | TI ( ((mental* or psychiatric* or psychological*) N3 (wellbeing or well-being)) ) OR AB ( ((mental* or psychiatric* or psychological*) N3 (wellbeing or well-being)) ) | 18,153 |
| S5 | TI ( ((mental* or psychiatric* or psychological*) N3 (health or disorder* or illness* or ill or problem* or disease*)) ) OR AB ( ((mental* or psychiatric* or psychological*) N3 (health or disorder* or illness* or ill or problem* or disease*)) ) | 212,972 |
| S4 | (MH "Wellness") | 8,845 |
| S3 | (MH "Psychological Well-Being") | 39,512 |
| S2 | (MH "Mental Disorders") OR (MH "Mental Disorders, Chronic") | 70,626 |
| S1 | (MH "Mental Health") | 60,247 |

### Ebsco Education Source

| **#** | **Query** | **Results** |
| --- | --- | --- |
| S23 | S21 OR S22 | 896 |
| S22 | S5 AND S13 AND S20 | 875 |
| S21 | S1 AND S13 | 49 |
| S20 | S14 OR S15 OR S16 OR S17 OR S18 OR S19 | 905,255 |
| S19 | TI ( ((vocational or trade*) N2 (school* or training or student* or studies or education or institution* or program*)) ) OR AB ( ((vocational or trade*) N2 (school* or training or student* or studies or education or institution* or program*)) ) | 22,745 |
| S18 | TI ( ((graduate or undergrad* or under-grad* or doctora* or phd or master* or postgraduate*) N2 (studies or education or institution* or student* or program* or course* or cohort* or training)) ) OR AB ( ((graduate or undergrad* or under-grad* or doctora* or phd or master* or postgraduate*) N2 (studies or education or institution* or student* or program* or course* or cohort* or training)) ) | 105,465 |
| S17 | TI tertiary education OR AB tertiary education | 3,642 |
| S16 | TI ( (higher education or higher learning) ) OR AB ( (higher education or higher learning) ) | 165,384 |
| S15 | TI ( (universit* or college* or campus*) ) OR AB ( (universit* or college* or campus*) ) | 755,226 |
| S14 | ((((DE "Universities & colleges") AND (DE "Postsecondary education" OR DE "Higher education" OR DE "Vocational education" OR DE "Vocational schools")) AND (DE "Graduate education" OR DE "Postdoctoral programs" OR DE "Doctoral programs" OR DE "College students" OR DE "Graduate students" OR DE "Undergraduates" OR DE "Vocational school students" OR DE "Undergraduate programs")) OR (DE "College campuses")) OR (DE "Doctoral students") | 9,770 |
| S13 | S6 OR S7 OR S8 OR S9 OR S10 OR S11 OR S12 | 177,445 |
| S12 | TI shared decision making OR AB shared decision making | 1,452 |
| S11 | TI partnership approach* OR AB partnership approach* | 786 |
| S10 | TI ( (student* N4 (partner* or creat* or produc* or collab* or design* or engage* or drive* or change agent* or knowledge user* or oriented or centered or centred or advisor*)) ) OR AB ( (student* N4 (partner* or creat* or produc* or collab* or design* or engage* or drive* or change agent* or knowledge user* or oriented or centered or centred or advisor*)) ) | 113,067 |
| S9 | TI ( (colead or cocreat* or codesign* or coresearch* or coenquire* or coproduce*) ) OR AB ( (colead or cocreat* or codesign* or coresearch* or coenquire* or coproduce*) ) | 539 |
| S8 | TI ( (co N0 (lead or creat* or design* or research* or enquire* or produce*)) ) OR AB ( (co N0 (lead or creat* or design* or research* or enquire* or produce*)) ) | 3,637 |
| S7 | TI ( ((collab* or inclusi* or include* or participa* or action or community based or peer or communit* or together or team*) N3 research*) ) OR AB ( ((collab* or inclusi* or include* or participa* or action or community based or peer or communit* or together or team*) N3 research*) ) | 62,878 |
| S6 | DE "Action research" OR DE "Action research in education" | 7,352 |
| S5 | S2 OR S3 OR S4 | 72,446 |
| S4 | TI wellness OR AB wellness | 5,655 |
| S3 | TI ( ((mental* or psychiatric* or psychological*) N3 (wellbeing or well-being)) ) OR AB ( ((mental* or psychiatric* or psychological*) N3 (wellbeing or well-being)) ) | 6,683 |
| S2 | TI ( ((mental* or psychiatric* or psychological*) N3 (health or disorder* or illness* or ill or problem* or disease*)) ) OR AB ( ((mental* or psychiatric* or psychological*) N3 (health or disorder* or illness* or ill or problem* or disease*)) ) | 63,432 |
| S1 | DE "Mental health of black college students" OR DE "Mental health of college students" | 1,114 |

### ProQuest ERIC

(MAINSUBJECT.EXACT("Mental Health") OR (noft(((mental or mentally or psychiatric or psychological or psychologically) NEAR/3 (health or disorder or disorders or disordered or illness or illnesses or ill or problem or problems or disease or diseases))) OR noft(((mental or mentally or psychiatric or psychological or psychologically) NEAR/3 (wellbeing or well-being))) OR noft(wellness))) AND (MAINSUBJECT.EXACT("Action Research") OR (noft(((collab* OR inclusi* OR include* OR participa* OR action OR "community based" OR peer OR communit* OR together OR team*) NEAR/3 research*)) OR noft((co NEAR/1 (lead OR creat* OR design* OR research* OR enquire* OR produce*))) OR noft((colead OR cocreat* OR codesign* OR coresearch* OR coenquire* OR coproduce*)) OR noft((student* NEAR/4 (partner* OR creat* OR produc* OR collab* OR design* OR engage* OR drive* OR "change agent*" OR "knowledge user*" OR oriented OR centered OR centred OR advisor*))) OR noft(partnership approach*) OR noft(shared decision making))) AND ((MAINSUBJECT.EXACT("Higher Education") OR MAINSUBJECT.EXACT("Undergraduate Study") OR MAINSUBJECT.EXACT("Postdoctoral Education") OR MAINSUBJECT.EXACT("College Students") OR MAINSUBJECT.EXACT("Doctoral Programs") OR MAINSUBJECT.EXACT("Graduate Study") OR MAINSUBJECT.EXACT("Community Colleges") OR MAINSUBJECT.EXACT("Colleges") OR MAINSUBJECT.EXACT("College Programs") OR MAINSUBJECT.EXACT("Masters Programs") OR MAINSUBJECT.EXACT("Vocational Schools") OR MAINSUBJECT.EXACT("Undergraduate Students") OR MAINSUBJECT.EXACT("Universities") OR MAINSUBJECT.EXACT("Graduate Students") OR MAINSUBJECT.EXACT("Postsecondary Education")) OR (noft(("post-secondary" OR postsecondary OR universit* OR college* OR campus* OR "higher education" OR "higher learning" OR "tertiary education")) OR noft(((graduate or undergrad* or under-grad* or doctora* or phd or master* or postgraduate*) NEAR/2 (studies or education or institution* or student* or program* or course* or cohort* or training))) OR noft(((vocational or trade*) NEAR/2 (school* or training or student* or studies or education or institution* or program*)))))

=1257

### ProQuest International Bibliography of the Social Sciences (IBSS)

((MAINSUBJECT.EXACT("Mental health") OR MAINSUBJECT.EXACT("Mental disorders")) OR (noft(((mental or mentally or psychiatric or psychological or psychologically) NEAR/3 (health or disorder or disorders or disordered or illness or illnesses or ill or problem or problems or disease or diseases))) OR noft(((mental or mentally or psychiatric or psychological or psychologically) NEAR/3 (wellbeing or well-being))) OR noft(wellness))) AND (MAINSUBJECT.EXACT("Action Research") OR noft(((collab* OR inclusi* OR include* OR participa* OR action OR "community based" OR peer OR communit* OR together OR team*) NEAR/3 research*)) OR noft((co NEAR/1 (lead OR creat* OR design* OR research* OR enquire* OR produce*))) OR noft((colead OR cocreat* OR codesign* OR coresearch* OR coenquire* OR coproduce*)) OR noft((student* NEAR/4 (partner* OR creat* OR produc* OR collab* OR design* OR engage* OR drive* OR "change agent*" OR "knowledge user*" OR oriented OR centered OR centred OR advisor*))) OR noft(partnership approach*) OR noft(shared decision making)) AND ((MAINSUBJECT.EXACT("Community colleges") OR MAINSUBJECT.EXACT("College students") OR MAINSUBJECT.EXACT("Graduate students") OR MAINSUBJECT.EXACT("Graduate studies") OR MAINSUBJECT.EXACT("University students") OR MAINSUBJECT.EXACT("Vocational education") OR MAINSUBJECT.EXACT("Colleges & universities") OR MAINSUBJECT.EXACT("Community college education") OR MAINSUBJECT.EXACT("Higher education")) OR (noft(("post-secondary" OR postsecondary OR universit* OR college* OR campus* OR "higher education" OR "higher learning" OR "tertiary education")) OR noft(((graduate or undergrad* or under-grad* or doctora* or phd or master* or postgraduate*) NEAR/2 (studies or education or institution* or student* or program* or course* or cohort* or training))) OR noft(((vocational or trade*) NEAR/2 (school* or training or student* or studies or education or institution* or program*)))))

=1005

### ProQuest Sociological Abstracts

( MAINSUBJECT.EXACT("Mental Health") OR noft(((mental or mentally or psychiatric or psychological or psychologically) NEAR/3 (health or disorder or disorders or disordered or illness or illnesses or ill or problem or problems or disease or diseases))) OR noft(((mental or mentally or psychiatric or psychological or psychologically) NEAR/3 (wellbeing or well-being))) OR noft(wellness)) AND ( MAINSUBJECT.EXACT("Action Research") OR noft(((collab* OR inclusi* OR include* OR participa* OR action OR "community based" OR peer OR communit* OR together OR team*) NEAR/3 research*)) OR noft((co NEAR/1 (lead OR creat* OR design* OR research* OR enquire* OR produce*))) OR noft((colead OR cocreat* OR codesign* OR coresearch* OR coenquire* OR coproduce*)) OR noft((student* NEAR/4 (partner* OR creat* OR produc* OR collab* OR design* OR engage* OR drive* OR "change agent*" OR "knowledge user*" OR oriented OR centered OR centred OR advisor*))) OR noft(partnership approach*) OR noft(shared decision making) ) AND ( (MAINSUBJECT.EXACT("Community Colleges") OR MAINSUBJECT.EXACT("Graduate Students") OR MAINSUBJECT.EXACT("Vocational Education") OR MAINSUBJECT.EXACT("Doctoral Programs") OR MAINSUBJECT.EXACT("Postdoctoral Programs") OR MAINSUBJECT.EXACT("Undergraduate Students") OR MAINSUBJECT.EXACT("Polytechnic Schools") OR MAINSUBJECT.EXACT("Graduate Schools") OR MAINSUBJECT.EXACT("Higher Education") OR MAINSUBJECT.EXACT("Masters Programs") OR MAINSUBJECT.EXACT("Colleges") OR MAINSUBJECT.EXACT("College Students") OR MAINSUBJECT.EXACT("Universities") OR MAINSUBJECT.EXACT("Undergraduate Programs")) OR noft(("post-secondary" OR postsecondary OR universit* OR college* OR campus* OR "higher education" OR "higher learning" OR "tertiary education")) OR noft(((graduate or undergrad* or under-grad* or doctora* or phd or master* or postgraduate*) NEAR/2 (studies or education or institution* or student* or program* or course* or cohort* or training))) OR noft(((vocational or trade*) NEAR/2 (school* or training or student* or studies or education or institution* or program*))) )

=1109
